# Cross-Sectional Study on the Influence of Religion on the Consumption of Ultra-Processed Food in Spanish Schoolchildren in North Africa

**DOI:** 10.3390/nu17020251

**Published:** 2025-01-11

**Authors:** Miriam Mohatar-Barba, Emilio González-Jiménez, María López-Olivares, Ángel Fernández-Aparicio, Jacqueline Schmidt-RioValle, Carmen Enrique-Mirón

**Affiliations:** 1 Department of Nursing, Faculty of Health Sciences, Melilla Campus, University of Granada, 52005 Melilla, Spain; miriamb@ugr.es; 2 Instituto de Investigación Biosanitaria (ibs.GRANADA), 18016 Granada, Spain; 3 Department of Nursing, Faculty of Health Sciences, University of Granada, 18016 Granada, Spain; anfeapa@ugr.es (Á.F.-A.); jschmidt@ugr.es (J.S.-R.); 4 Department of Nutrition and Food Science, Faculty of Health Sciences, Melilla Campus, University of Granada, 52005 Melilla, Spain; mlopezolivares@ugr.es; 5 HUM-613 Research Group, Department of Inorganic Chemistry, Faculty of Health Sciences, Melilla Campus, University of Granada, C/Santander s/n, 52005 Melilla, Spain; cenrique@ugr.es

**Keywords:** ultra-processed foods, adolescents, healthy habits, health promotion

## Abstract

(1) Background: The consumption of ultra-processed foods (UPFs) constitutes a public health problem given their high availability and easy accessibility among children and young people and their influence on the development of non-communicable diseases in the long term. In this context, culture and religion may be modulating factors for the consumption of processed food. The aim of this study is to assess the consumption of UPFs in Spanish schoolchildren living in Melilla (North Africa), together with the possible impact of religion on this. (2) Methods: A cross-sectional study of 590 Christian and Muslim schoolchildren aged 15–17 years was conducted. The NOVA food classification was used to identify UPFs. Associations between religion and daily consumption were identified using risk analysis (Odds Ratio). (3) Results: Muslim schoolchildren had a higher consumption of industrial juices [OR = 2.700, 95%CI = 1.830–4.037], milkshakes [OR = 2.925, 95% = 1.850–4.748], industrial pastries [OR = 2.217, 95% = 1.440–3.510], sweets [OR = 2.197, 95%CI = 1.437–3.541], chocolates [OR = 2.272, 95%CI = 1.482–3.606] and savory snacks [OR = 3.431, 95%CI = 1.844–6.579] compared to that observed among Christians. (4) Conclusions: Both Muslim and Christian schoolchildren had a high consumption of UPFs. Regarding the potential impact of religion on the consumption of UPF, we observed that Muslim schoolchildren consumed three to four times more UPF than Christian schoolchildren. These results show a shift away from a healthy eating pattern, especially among Muslim schoolchildren. Thus, it is necessary to implement nutritional education strategies in order to understand and control the consumption of UPF in adolescents, thereby reducing the occurrence of non-communicable diseases in the long term.

## 1. Introduction

Adolescence is a developmental stage in which inadequate eating practices are frequently observed. Many adolescents skip meals, do not maintain a regular schedule for eating, and often consume hypercaloric, low nutritional, and highly processed foods [1]. These habits represent a departure from the Mediterranean dietary pattern, mainly due to globalization, thus imposing an eminently Western dietary model [2].

These changes reflect the impact of recent global factors, such as the COVID-19 pandemic, which has profoundly altered behavioral patterns. During confinement, increased sedentary lifestyles and changes in daily routines contributed to a deterioration in eating habits in many adolescents, favoring the consumption of hypercaloric and non-nutritious foods [3]. In addition, the interruption of face-to-face school activities and the closure of recreational spaces led to greater social isolation and dependence on fast food and ultra-processed snacks as a way of coping with stress and uncertainty [4].

Despite advertising campaigns on the importance of healthy eating and lifestyles, in developed countries, the availability and easy access to these food products has contributed to the deterioration of the quality of the diet and the health of the young population [5,6].

The consumption of ultra-processed foods (UPFs) is increasing and becoming more prevalent among the world’s population. Although the consumption of UPF is higher in high-income countries, an increase in consumption is observed in low-income countries, influenced by economic factors, where price is an important element in the choice of product [7]. Thus, the population of countries such as the United States, Canada, Chile, and European countries such as France and Germany has increased the consumption of UPFs, especially when the purchase is made in supermarkets as opposed to small stores where fresh and natural foods predominate [8,9].

Nutritionally, UPFs are characterized by low amounts of fiber, protein, and micronutrients while being energy-dense and high in saturated and trans fatty acids, added sugars, and sodium [10,11,12]. In addition, their composition usually contains flavoring substances, colorants, emulsifiers, and other additives that enhance their sensory characteristics by giving them a good appearance, smell, and taste, thus creating low-cost, hyperpalatable products that are very attractive to the adolescent population. These characteristics of UPF can favor the development of non-communicable diseases (NCDs), such as type-2 diabetes mellitus (DM2), metabolic syndrome (MetS), obesity, cardiovascular diseases, and cancer [13]. Studies such as the one developed by Kelly and Jacoby [14] demonstrate a close relationship between the consumption of UPFs and the onset of disorders such as obesity at increasingly younger ages.

Although there is currently evidence of a decrease in childhood obesity in developed countries, Spain continues to be the second country in Europe with the highest prevalence of overweight and obesity in children and young people [15]. Thus, according to the latest study conducted by the Spanish Agency for Food Safety and Nutrition [16], one in three children between 2 and 17 years of age (19.2%) are overweight and one in ten are obese (10.7%). In this context, adolescence, in addition to being a key period due to physiological and psychological changes, is a stage in which eating habits can be conditioned by other factors such as culture or the religious group to which an individual belongs [17].

In this sense, Melilla, a Spanish city located on the northwest coast of Africa where citizens of different cultures and religions have lived together for centuries and with an important Christian and Muslim student community, offers a unique opportunity to investigate whether this cultural and religious diversity has an impact on the dietary preferences of schoolchildren [18,19].

Given the increasing prevalence of UPF consumption among adolescents and its associated health risks, it is essential to understand the factors that influence these dietary habits. While previous studies have extensively analyzed the nutritional impact of UPF and its correlation with NCDs, there is a lack of research addressing the role of cultural and religious diversity in shaping these food consumption patterns [20]. Melilla, with its unique demographic characteristics, represents a valuable setting in which to investigate this relationship. The need for this study arises from a twofold gap in the literature: the limited understanding of how religion influences eating habits in multicultural settings and the absence of data specifically targeting adolescents in Melilla, as this city is often excluded from epidemiological studies conducted at the national level on these aspects. By addressing these gaps, this research contributes to expanding knowledge on culturally sensitive dietary interventions and public health strategies.

Therefore, this study aims to evaluate the consumption of ultra-processed products among a sample of Spanish Christian and Muslim schoolchildren, investigating the possible influence of religion on food consumption patterns.

## 2. Materials and Methods

### 2.1. Study Design and Subjects

A cross-sectional study was carried out on a population of 590 schoolchildren (233 males and 357 females), all of whom were in compulsory secondary education and belonged to 6 educational centers in the Autonomous City of Melilla. The participants were selected by purposive sampling and had a mean age of 15.65 ± 0.73 years.

### 2.2. Data Collection

**Figure 1:**
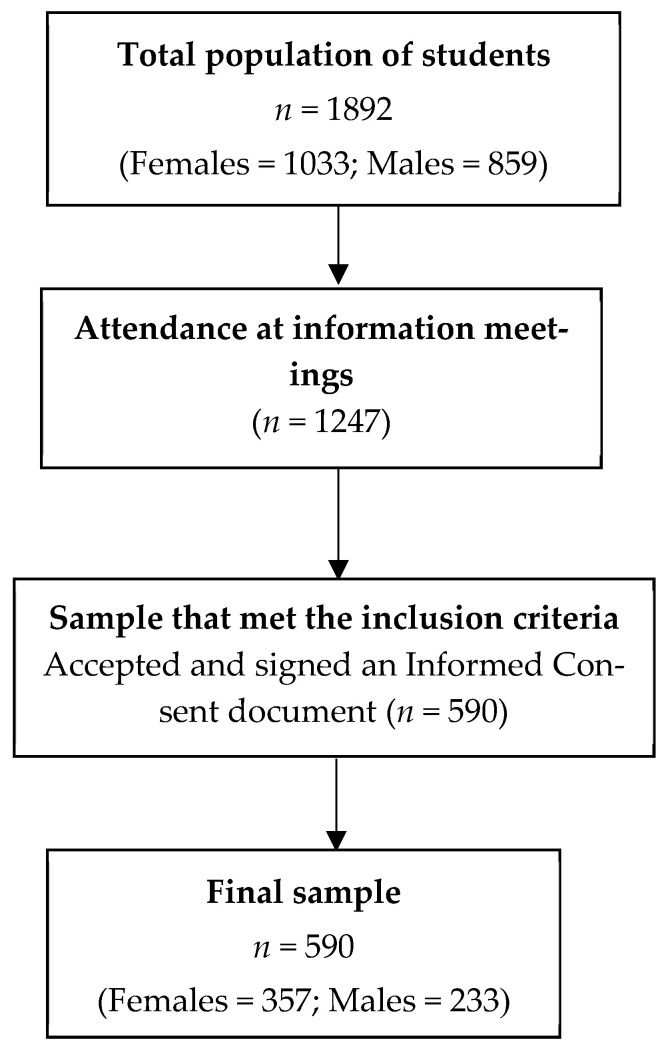
Flow diagram of the recruitment process.

To participate in the study, a letter of invitation was sent to the directors of the 6 Secondary Education Centers, all of which accepted to participate. In each Educational Center, Christian and Muslim students between 15 and 17 years of age (corresponding to the 3rd and 4th years of Compulsory Secondary Education (ESO) and the 1st year of Baccalaureate education) were invited to take part in the study. Prior to their inclusion, parents or legal guardians had to sign the Informed Consent document authorizing their child’s participation in the research. Figure 1 summarizes the recruitment process. The study reports conform to STROBE’s general guidelines [21].

In September 2023, informational meetings were held with the parents or legal guardians of each school. At these meetings, parents or legal guardians were informed about the assessments and questionnaires that their children would have to complete to participate in the study. In October 2023, each participant underwent an anthropometric assessment and a body composition analysis, and their eating habits were evaluated.

The study was approved by the Research Ethics Committee of the University of Granada (Code 2752/CEIH/2022 approved on 8 November 2022). In addition, it was authorized by the Dirección Provincial de Educación del Ministerio de Educación, Formación Profesional y Deporte de Melilla. All parents or legal guardians of the participating schoolchildren signed the Informed Consent document, and the confidentiality of their personal information was guaranteed by coding the data. This research was conducted in strict compliance with the International Code of Medical Ethics established by the World Medical Association and the Declaration of Helsinki [22].

### 2.3. Blood Pressure

The participants’ blood pressure (BP) was measured with a pre-calibrated sphygmomanometer and a Littmann^®^ stethoscope following the recommendations of the Professional and Public Education Subcommittee of the High Blood Pressure Research Council of the American Heart Association [23]. During BP measurement, the subject had to remain seated in a chair for 5 min with the back supported, feet flat on the floor, and wrist relaxed at the level of the heart. Participants were also required to remain relaxed and silent.

### 2.4. Dietary Intake

Each participant completed a food frequency questionnaire (FFQ) consisting of 44 foods designed to assess the frequency of consumption of each food daily, weekly, or monthly [24]. In addition, a 72 h food record was used that recorded the foods eaten on three days (Thursday, Friday, and Saturday) to identify possible variations in consumption frequency between weekdays and weekends [25].

The completion of this registry was carried out by trained researchers and through face-to-face interviews, in which the participants were asked for information on the foods, beverages, and supplements ingested during the three days mentioned. In addition, to facilitate the estimation of quantities, standardized household measurements and food images were used in the interview.

For the estimation of energy intake and calculation of macronutrients and micronutrients, the nutritional calculator offered by the Institute of Endocrinology and Nutrition of Valladolid (IENVA) was used “https://calcdieta.ienva.org/tu_menu.php” (accessed on 1 February 2024). 

On the other hand, the NOVA classification was used to categorize the foods and beverages included in the FFQ and in the 72 h log, assigning them to one of four groups according to their degree of industrial processing and the purpose of the processing. This classification establishes four groups of foods based on their degree of processing: group 1 describes unprocessed or minimally processed foods, such as fruits, vegetables, milk, and meat; group 2 comprises processed culinary ingredients, which are substances extracted from foods for use in culinary preparation, cooking, and/or seasoning of group 1 foods, such as table salt, sugar, vegetable oils, and butter; group 3 includes processed products obtained by adding salt, sugar or another group 2 ingredients to group 1 foods, such as bread, fresh cheese, or canned vegetables; group 4 includes all ultra-processed foods (UPFs), which are mixtures of multiple industrial ingredients (e.g., sugar or another group 2 ingredients), e.g., high-fructose corn syrup), often with additives, such as colorants, emulsifiers, and preservatives, that disguise any undesirable sensory properties of the final product. Within this group, sugar-sweetened beverages, frozen meals, and packaged breads are included. For the 72 h log, we classified each food according to the NOVA classification and calculated the calories and key nutrients from the UPF consumed by each participant using the IENVA calculator.

### 2.5. Anthropometric Measurements

An anthropometric evaluation was carried out on all participants, including the determination of height, weight, body mass index (BMI), hip and waist circumference, as well as the waist–hip index (WHI) and waist–height index (WHI). For the determination of these anthropometric parameters, the standards defined by the International Society for the Advancement of Kinanthropometry (ISAK) [26] were followed.

Body weight and BMI were measured by electrical bioimpedance using a TANITA^®^ body composition analyzer, model SC-330, with participants being asked to remove their shoes beforehand. The measurements were performed by the same trained researcher first thing in the morning. Fat mass, lean mass, and muscle mass were also estimated using this body composition analyzer.

The height of the participants was measured using a SECA stadiometer (model 214) with an accuracy of 1 cm. For the determination of height, the participant had to stand with their back in contact with the stadiometer, with the heels together and the head oriented in the Frankfurt plane.

Waist circumference was measured in the horizontal plane at the point equidistant between the last rib and the upper edge of the iliac crest at the end of an exhalation. Hip circumference was measured at the maximum width of the buttocks. For both measurements, a Seca^®^ roll-up tape measure was used, with an accuracy of 1 mm, while the participant remained standing and in an anatomical position.

### 2.6. Other Variables

Another variable examined was religion, for which each participant self-identified his or her religious practice (Islam or Christianity). To measure this variable, the Religious Attitudes Questionnaire developed and validated by Orozco-Parra and collaborators [27] was used.

### 2.7. Statistical Analysis

The statistical analysis carried out was aimed at comparing Christian and Muslim schoolchildren in various aspects. In relation to the physical and sociodemographic characteristics of the participants, the mean ± standard deviation was used to express the continuous variables, whereas frequencies and percentages were used for the categorical variables. The non-normality of the quantitative variables (Shapiro–Wilk test) has led to the use of the Mann–Whitney U test.

Qualitative variables were analyzed using the Chi-square test or Fischer’s exact test. The number of participants who consumed ultra-processed foods (UPFs) at different times was assessed by the Chi-square test, and the frequency (percentage) was reported.

Given the dispersion of schoolchildren’s responses, the frequency of consumption among participating Christian and Muslim students was assessed by applying the Mann–Whitney U Ranks test for independent samples, expressed as the median and interquartile range. Odds Ratios were calculated by binary logistic regression analysis, taking Christian schoolchildren as the reference and adjusted for religion, with 95% confidence intervals (CIs).

To estimate the intake of UPFs (low intake or high intake), the intakes recommended by the World Health Organization (WHO) [28] were used. Statistical analyses were performed with SPSS version 28 [29], and significance was set at *p* < 0.05.

## 3. Results

Of the 590 schoolchildren participating in the study, 192 self-identified as Christians (86 boys and 106 girls), and 398 identified as Muslims (147 boys and 251 girls). All of them were students at public schools in the Autonomous City of Melilla.

### 3.1. Sociodemographic and Physical Characteristics of Participating Students

Table 1 shows the sociodemographic and physical characteristics together with the nutritional status and blood pressure levels of the schoolchildren participating in the study.

Statistically significant differences (p< 0.05) were found for the individual’s age, body mass index, nutritional status, waist circumference, and blood pressure. Regarding the nutritional status of the schoolchildren, there was a higher proportion of overweight and obesity in Christian males (31.4%) compared to Muslims. Regarding blood pressure levels, Muslim females were prehypertensive compared to Christian females (p < 0.040).

### 3.2. Schoolchildren Consuming Ultra-Processed Food (UPF) According to Sex and Religious Group

Table 2 shows the number of schoolchildren who consumed UPFs on a daily basis according to sex and religion. There are statistically significant differences in consumption between Christian (*p* < 0.05) and Muslim (*p* < 0.001) schoolchildren for certain UPFs such as industrial juices, sugary drinks, milkshakes, pastries, and sweets, among others, with higher consumption among Muslim schoolchildren.

Among the most consumed UPFs, Muslim men consumed more industrial juices (43.5%), followed by industrial sauces compared to Christian men. With respect to Muslim women, higher consumption of industrial juices (43%), industrial sauces (36.4%), and sweets (35.1%) were observed compared to Christian women. However, men and women who self-identify as Christians consume more sausages (27.9% and 24.5%, respectively) compared to Muslim men and women. On the other hand, sausages, regardless of whether fresh or cooked, such as frankfurters, bratwurst, or breakfast sausages, and hamburgers are the least consumed UPF in both sexes and religions, with very low percentages (5.9% and 9.2%, respectively).

### 3.3. Data on Energy and Key Nutrients Derived from the Ingestion of Ultra-Processed Food (UPF)

**Figure 2:**
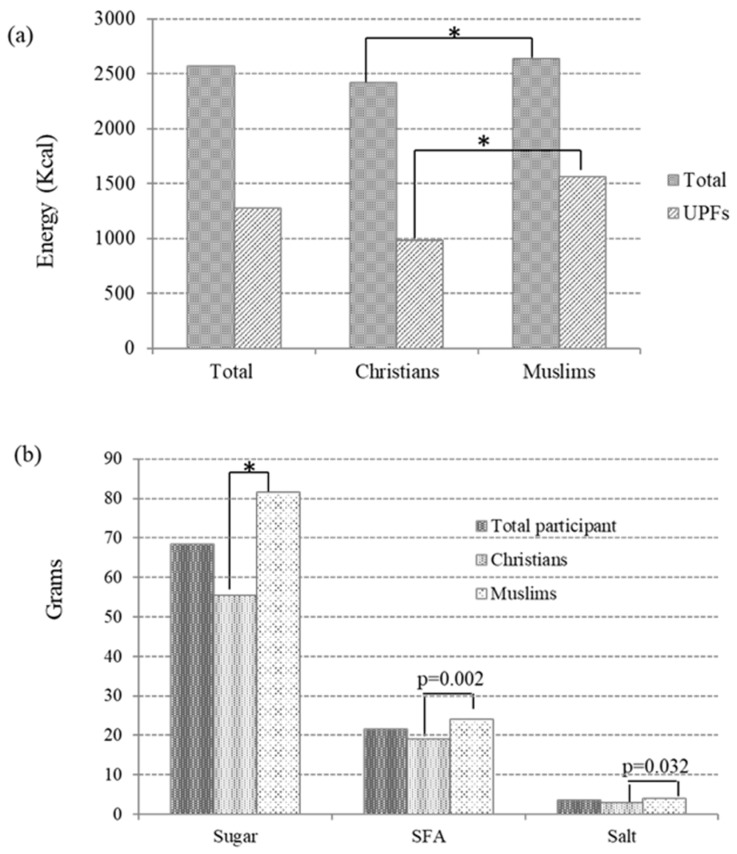
Data on total energy and ultra-processed food energy (**a**) and on the consumption of key nutrients from UPFs (**b**). UPFs, ultra-processed foods; SFA, saturated fatty acids. * *p* < 0.001.

Figure 2 shows the data related to total energy and energy derived from the consumption of UPF, as well as the data related to the most relevant nutrients linked to the consumption of UPF (sugar, SFA, and salt) according to religion. Daily intake from UPFs was 40.5% and 59.1% for Christians and Muslims, respectively. Statistically significant differences were appreciated for all data between Christian and Muslim participants.

### 3.4. Frequency of Consumption of Ultra-Processed Food (UPF) Among Christian and Muslim Schoolchildren

Table 3 shows the frequency of consumption of UPFs among the Christian and Muslim schoolchildren participating in the study. A higher consumption of industrial juices, milkshakes, industrial pastries, sweets, chocolate, potato chips, salty snacks, and industrial sauces was observed among Muslim schoolchildren (*p* < 0.001) compared to Christians. Likewise, there was also a higher consumption of sugary drinks among Muslim schoolchildren (*p* = 0.003) compared to Christians. As for sausages, consumption was higher among Christian schoolchildren compared to Muslim schoolchildren (*p* = 0.045).

### 3.5. Influence of Religious Groups on the Consumption of Ultra-Processed Food (UPF)

Table 4 shows the positive association between belonging to the Muslim religion and a higher frequency of consumption of UPFs. The highest Odds Ratio values observed were for the consumption of salty snacks [OR = 3.431, 95%CI = 1.844–6.579], industrial sauces [OR = 3.431, 95%CI = 1.635–3.704], smoothies [OR = 2.925, 95% = 1.850–4.748], and industrial juices [OR = 2.700, 95%CI = 1.830–4.037].

## 4. Discussion

To our knowledge, our study is a pioneer in evaluating the possible influence of religion on the consumption of ultra-processed food (UPF) in Spanish Christian and Muslim schoolchildren. As reflected in our results, the consumption of UPFs was high in both Muslim and Christian students, with the frequency of daily consumption of most UPFs being higher among Muslim schoolchildren, with the consumption of these foods being two to three times higher than that observed among Christian schoolchildren. These results show a departure from the healthy eating pattern, especially among Muslim schoolchildren.

Regarding nutritional status, our results reflect a higher prevalence of being overweight among Christian and Muslim males compared to females. Although our results show that Christian schoolchildren have a worse nutritional status, it is the Muslim schoolchildren who have a higher consumption of UPFs, with the consequent risk of developing obesity, metabolic disorders, mental disorders, and cardiovascular disease [30]. These results are similar to those proposed by El Mokhatari [31], who, in their study of adolescent immigrants from Northern Morocco, found that religious customs condition the choice of food, which can lead to the worsening of their nutritional status and favor the development of obesity.

On the other hand, our study highlights a higher rate of prehypertension among Muslim women compared to Christian women. Several recent studies address the prevalence of prehypertension in adolescents, especially in young women. The study by Thapa et al. [32], with a Nepalese adolescent population, found a prehypertension rate of 20.8%. For their part, Prasad et al. [33], in their study conducted in Pakistan, found that 25.8% of the female participants had prehypertension compared to 20.3% of the males, highlighting that hormonal factors and stress could influence these results. Similarly, more than half of the sample of adolescent participants in the longitudinal study by Israeli et al. [34] presented with prehypertension, and these figures increased with age. These findings underscore the need to pay special attention to prehypertension at an early age, and it is necessary to implement healthy lifestyles through educational strategies as a basis for preventing the future development of hypertension and cardiovascular events [35,36].

With regard to the frequency of consumption of UPFs, both Muslim and Christian schoolchildren had a high daily consumption of UPF. However, among Muslim schoolchildren, an increase in the frequency of the daily consumption of these products was observed, especially for industrial juices, sugary drinks, milkshakes, industrial pastries, sweets, chocolate, potato chips, salty snacks, and industrial sauces. These findings are consistent with those described by other studies whose authors describe an increasing consumption of UPF among the adolescent population, which raises the alarm for long-term health implications [37,38]. In Spain, Reales-Moreno et al. [39], in their study with adolescents, observed a frequency of UPF consumption of 7.72 times per day, mainly among males. This pattern of high consumption of UPFs was associated with a higher rate of depressive symptoms and other mental health problems in the entire sample.

Spain currently has a Nutrition, Physical Activity, and Obesity Prevention Strategy (known as the NAOS Strategy) [40], which is a program based on the promotion of health and prevention of diseases such as obesity and NCDs, through the promotion of healthy eating and the regular practice of physical exercise. In addition to counting on the collaboration of the different Autonomous Communities, the NAOS Strategy involves the whole of society in an attempt to raise awareness of the advantages of a healthy diet based on a reduced intake of UPF, as well as the health benefits derived from the practice of physical activity. However, studies such as those mentioned above or that by Sandri et al. [41] tend to only include a small percentage of young participants who adopt a plant-based diet, have healthier lifestyle patterns, and consume fewer UPFs; the study by Romero et al. [42] states that Spanish youth are the highest consumers of ultra-processed foods, contributing around 24–31% to their total dietary energy, demonstrating that educational programs do not seem to have had sufficient impact in reducing UPF consumption among the youth population.

For their part, Valizadeh and Weng [43] suggest that establishing a tax on UPFs, together with financial aid for the purchase of minimally processed foods, could be an effective social policy to promote healthier food choices, especially in low-income countries. In Spain, thanks to the latest plan of the Ministry of Health, Consumption and Social Welfare, a tax on UPFs could be an effective social policy to promote healthier food choices, especially in low-income countries [44]; for instance, as part of the “Collaborative plan for the improvement of the composition of food and beverages and other measures 2017–2020”, the composition of more than 3000 food products has been modified, achieving a 10% reduction in their salt and sugar content and a between 5 and 10% reduction in saturated fats. However, its social and health impact is limited because its application is not mandatory for the entire food sector.

In our study, all the UPFs analyzed contained high amounts of saturated fatty acids. In this sense, although Muslim schoolchildren, for religious reasons, consume fewer sausages, in their case, the intake of saturated fatty acids comes from the rest of the UPFs that they consume with high frequency. From these results, it can be said that the consumption of UPFs among the school population studied seems to be influenced, among other factors, by religion. These findings have clinical and social relevance, as they justify the need to create educational programs focused on promoting healthy eating patterns from an early age, with the necessary involvement of the family environment, always taking into account the sociocultural and religious environment of adolescents [45,46,47].

This study has strengths and limitations. Among this study’s strengths is its pioneering nature, being the first study to analyze the possible influence of religion on the consumption of UPFs among Spanish schoolchildren. Our findings should be considered for implementation in larger studies in different geographic contexts, which can allow us to achieve more valid results. One limitation of this study is the unequal distribution of the sample according to religious groups, with more Muslim than Christian adolescents participating. This purposive sampling design was chosen to focus on specific subgroups, which may limit the generalizability of our findings to the adolescent population at large. The disproportionate representation of Muslim adolescents could introduce selection bias, potentially influencing the results. We acknowledge this limitation and suggest that future studies seek a more balanced distribution to explore whether these results hold across religious groups. In addition, sensitivity analyses could be conducted to assess whether the imbalance in the sample affected the results.

On the other hand, regarding future implications, despite existing campaigns, UPF consumption remains high in adolescents, suggesting that new and more effective educational strategies are needed. Future research could evaluate the effectiveness of specific nutritional interventions in schools and communities to reduce the consumption of these foods, with a particular focus on social networks and family education.

## 5. Conclusions

In conclusion, both Muslim and Christian schoolchildren showed a high intake of UPFs. However, Muslim schoolchildren showed a markedly higher intake in all subcategories of UPFs compared to their Christian peers, with a significantly higher daily consumption of salty snacks, industrial sauces, and industrial juices, among others. These results reveal that the eating habits of adolescents, especially among Muslims, deviate considerably from a healthy eating pattern.

Regarding the influence of religion on eating behaviors, Muslim adolescents were observed to consume UPFs at rates approximately two to three times higher than their Christian counterparts. This underscores the need to monitor the consumption of UPFs among school-aged children, both in healthcare settings and through culturally sensitive health promotion programs. Such initiatives should aim to address the sociocultural factors that influence these dietary patterns and prioritize early nutrition education to prevent conditions related to the excessive consumption of UPF, such as metabolic syndrome. Considering the long-term risks associated with high UPF consumption, including obesity and cardiovascular disease, is essential to effectively mitigate these health risks.

## Figures and Tables

**Table 1:** Sociodemographic and physical characteristics of the participating schoolchildren.

Variables			Males (n = 233)		Females (n = 357)	
		All (n = 590)	Christians (n = 86)	Muslims (n = 147)	Christians (n = 106)	Muslims (n = 251)
Age (years)		15.65 ± 0.73	15.88 ± 0.72	15.67 ± 0.76 *	15.63 ± 0.69	15.56 ± 0.72
Height (cm)		166.74 ± 9.33	174.31 ± 7.60	175.07 ± 7.07	161.20 ± 6.78	161.60 ± 6.09
Weight (kg)		63.18 ± 14.13	70.89 ± 16.98	67.01 ± 15.44	59.69 ± 10.78	59.77 ± 11.77
BMI (kg/m^2^)		22.72 ± 4.65	23.75 ± 6.47 *	21.77 ± 4.40	22.93 ± 3.99	22.83 ± 4.23
Nutritional status:						
	Low weight	82 (13.9)	8 (9.3)	36 (24.5) *	7 (6.6)	31 (12.4)
	Normal weight	347 (58.8)	51 (59.3)	75 (51.0)	69 (65.1)	152 (60.6)
	Overweight	117 (19.8)	17 (19.8) *	28 (19.0)	24 (22.6)	48 (19.1)
	Obesity	44 (7.5)	10 (11.6) *	8 (5.4)	6 (5.7)	20 (8.0)
WC (cm)		74.24 ± 10.62	79.28 ± 11.27	75.43 ± 10.46	71.77 ± 8.99	72.87 ± 10.50
HC (cm)		98.25 ± 35.40	107.46 ± 8.38 *	95.40 ± 10.93	96.51 ± 10.55	97.49 ± 12.00
WHR		0.771 ± 0.130	0.80 ± 0.11	0.79 ± 0.9	0.75 ± 0.13	076 ± 0.15
Waist-to-height ratio		0.45 ± 0.06	0.45 ± 0.06	0.43 ± 0.06	0.44 ± 0.06	0.45 ± 0.06
Fat Mass (Kg)		14.58 ± 9.03	12.26 ± 9.60	9.96 ± 8.10	17.12 ± 10.04	17.01 ± 7.54
Lean Mass (Kg)		48.54 ± 9.95	58.65 ± 9.20	56.53 ± 8.75	43.13 ± 5.24	42.67 ± 5.34
Muscle Mass (Kg)		46.56 ± 16.02	55.00 ± 9.67	53.74 ± 8.31	40.94 ± 4.98	41.84 ± 20.76
SBP (mmHg)		114.97 ± 15.74	124.20 ± 18.84	119.02 ± 15.98	115.73 ± 13.15	109.12 ± 12.95
DBP (mmHg)		70.67 ± 10.45	72.71 ± 10.72	70.26 ± 11.22	71.98 ± 10.66	69.66 ± 9.70
MBP		92.82 ± 11.06	98.46 ± 12.13	94.64 ± 11.30	93.86 ± 9.92	89.39 ± 9.86
Blood pressure classification:						
	Normal	537 (91.0)	67 (34.4)	128 (65.5)	103 (30.1)	239 (69.9) *
	Prehypertensive	36 (6.1)	13 (50.0)	13 (50.0)	0	10 (100) *
	Hypertensive	17 (2.9)	6 (50.0)	6 (50.0)	3 (60.0)	2 (40)

Data are mean values ± SDs and percentages. Abbreviations: BMI: body mass index; WC: waist circumference; HC: hip circumference; WHR: waist-to-hip ratio; SBP: systolic blood pressure; DBP: diastolic blood pressure; MBP: media blood pressure. Normal blood pressure: systolic (120–129 mmHg) and diastolic (80–84 mmHg); prehypertensive: systolic (130–139 mmHg) and diastolic (85–89 mmHg); hypertensive: systolic (≥140–159 mmHg) and diastolic (≥90–99 mmHg). Chi-square for categorical variables and Mann–Whitney U test for continuous variables. * *p* < 0.05. Christians vs. Muslims.

**Table 2:** Number of adolescents who consumed UPFs on a daily basis according to sex and religion.

		Males (n = 233)		Females (n = 357)	
	All (n = 590)	Christians (n = 86)	Muslims (n = 147)	Christians (n = 86)	Muslims (n = 147)
Industrial juices	214 (36.3)	20 (23.3)	64 (43.5) **	22 (20.8)	108 (43.0) **
Sweetened beverages	149 (25.3)	21 (24.4)	42 (28.6)	14 (13.2)	72 (28.7) **
Milkshakes	147 (24.9)	12 (14.0)	40 (27.2) **	13 (12.3)	82 (32.8) **
Industrial pastries	147 (24.9)	16 (18.6)	41 (27.9)	14 (13.2)	76 (30.3) **
Sweets	143 (24.2)	11 (12.8)	26 (17.7)	18 (17.0)	88 (35.1) **
Chocolate	149 (25.3)	16 (18.6)	39 (26.7)	14 (13.2)	80 (31.9) **
Potato chips (bag)	77 (13.1)	8 (9.3)	16 (10.9)	7 (6.6)	46 (18.3) **
Salty snacks	87 (14.7)	4 (4.7)	21 (14.3) *	8 (7.5)	46 (18.3) **
Industrial sauces	188 (31.9)	20 (23.3)	59 (40.1) *	18 (17.0)	91 (36.4) **
Sausages (fresh or cooked)	35 (5.9)	3 (3.5)	7 (4.8)	7 (6.6)	18 (7.2)
Sausages	111 (18.8)	24 (27.9) **	20 (13.7)	26 (24.5) *	41 (16.3)
Hamburgers	54 (9.2)	5 (5.8)	12 (8.2)	11 (10.4)	26 (10.4)

Note. Data are presented as the frequency and total percentage of subjects that consume each ultra-processed product. Chi-square test. * *p* < 0.05; ** *p* > 0.001.

**Table 3:** Frequency of consumption of UPFs among Christian and Muslim schoolchildren participating in the study.

		All (n = 590)	
	All (n = 590)	Christians (n = 192)	Muslims (n = 398)
Industrial juices	4 (4)	3 (3)	5 (4) **
Sweetened beverages	4 (4)	3 (4)	4 (4) *
Milkshakes	4 (4)	3 (4)	5 (4) **
Industrial pastries	4 (2)	4 (3)	5 (3) **
Sweets	4 (3)	4 (3)	5 (3) **
Chocolate	4 (4)	4 (3)	5 (3) **
Potato chips (bag)	4 (3)	4 (3)	5 (3) **
Salty snacks	4 (3)	4 (3)	5 (2) **
Industrial sauces	5 (3)	4 (3)	5 (3) **
Sausages (fresh or cooked)	2 (3)	2 (4)	2 (3)
Sausages	4 (3)	4 (3)	3 (4) *
Hamburgers	3 (3)	3 (3)	3 (3)

Note. Data are presented as median and interquartile range. Mann–Whitney U test. * *p* < 0.05; ** *p* > 0.001.

**Table 4:** The association of belonging to the Christian or Muslim religion and the frequency of consumption of UPF.

Ultra-Processed Products		n	%	OR	95% IC
Industrial juices					
	Low consumption < 1	376	63.7	1	
	High consumption > 1	214	36.3	2.700 **	1.830–4.037
Sugared beverages					
	Low consumption < 1	441	74.7	1	
	High consumption > 1	149	25.3	1.824 *	1.76–2.757
Milkshakes					
	Low consumption < 1	442	75.0	1	
	High consumption > 1	147	25.0	2.925 **	1.850–4.748
Industrial pastries					
	Low consumption < 1	443	75.1	1	
	High consumption > 1	147	24.9	2.217 **	1.440–3.510
Sweets					
	Low consumption < 1	447	75.8	1	
	High consumption > 1	143	24.2	2.197 **	1.437–3.541
Chocolate					
	Low consumption < 1	440	74.7	1	
	High consumption > 1	149	25.3	2.272 **	1.482–3.606
Potato chips (bag)					
	Low consumption < 1	513	86.9	1	
	High consumption > 1	77	13.1	2.176 *	0.630–2.140
Salty snacks					
	Low consumption < 1	503	85.3	1	
	High consumption > 1	87	14.7	3.431 **	1.844–6.579
Industrial sauces					
	Low consumption < 1	401	68.1	1	
	High consumption > 1	188	31.9	2.508 **	1.635–3.704
Sausages (fresh or cooked)					
	Low consumption < 1	555	94.1	1	
	High consumption > 1	35	5.9	1.184	0.574–2.594
Sausages					
	Low consumption < 1	479	81.2	1	
	High consumption > 1	111	18.8	0.511	1.071–1.530
Hamburgers					
	Low consumption < 1	536	90.8	1	
	High consumption > 1	54	9.2	1.117	0.630–2.140

Christians were taken as a reference. Data are presented as Odds Ratios (ORs) with 95% confidence intervals (CIs) using a logistic regression model. ORs adjusted for sex and religion. * *p* < 0.05; ** *p* < 0.001.

## Data Availability

The data presented in this study are available on request from the corresponding author. The data are not publicly available due to the need to maintain the privacy of participants.
